# Clinical Decision-Making and Imaging-Guided Follow-Up Strategies in Spontaneous Coronary Artery Dissection

**DOI:** 10.3390/jcdd13050190

**Published:** 2026-04-29

**Authors:** Koichi Nakamura, Osamu Kurihara, Daijirou Sonoda, Ayane Kobayashi, Kento Tani, Masayuki Tsutsumi, Hiroki Goda, Nobuaki Kobayashi, Masamichi Takano, Kuniya Asai

**Affiliations:** 1 Cardiovascular Medicine, Nippon Medical School Chiba Hokusoh Hospital, Inzai 270-1694, Japan; s13-079nk@nms.ac.jp (K.N.);; 2 Department of Cardiovascular Medicine, Nippon Medical School, Tokyo 113-8603, Japan; kasai@nms.ac.jp

**Keywords:** spontaneous coronary artery dissection, invasive coronary angiography, intravascular imaging, coronary computed tomography angiography

## Abstract

Spontaneous coronary artery dissection (SCAD) is an important non-atherosclerotic cause of acute coronary syndrome, predominantly affecting younger women without traditional cardiovascular risk factors. In hemodynamically stable patients, accumulating evidence supports a conservative management strategy owing to the high rate of spontaneous vessel healing, while technically challenging invasive interventions should be reserved for selected high-risk cases. Despite growing evidence regarding acute management, recurrent SCAD and other adverse cardiovascular events have been reported during follow-up, underscoring the need for surveillance. However, optimal strategies for post-acute follow-up and for assessing the appropriateness of treatment decisions remain insufficiently established. This review focuses on clinical decision-making in the management of SCAD, with particular emphasis on follow-up assessment. We summarize the existing evidence regarding indications for conservative versus invasive treatment and discuss the clinical rationale for longitudinal imaging surveillance. Special attention is given to the role of non-invasive follow-up using coronary computed tomography angiography, including confirmation of vessel healing, evaluation of residual intramural hematoma, and assessment of distal coronary flow. Given the heterogeneity of SCAD and the risk of recurrence, individualized treatment decisions and structured follow-up strategies are essential to optimize management, avoid unnecessary invasive procedures, and support care and risk stratification in patients with SCAD.

## 1. Introduction

Spontaneous coronary artery dissection (SCAD) has emerged over the past two decades as an important cause of acute coronary syndrome (ACS), particularly among younger and middle-aged women without traditional cardiovascular risk factors, and is now recognized as a leading cause of myocardial infarction in women around 50 years of age [1,2,3,4,5,6]. SCAD is characterized by spontaneous separation of the coronary arterial wall due to intramural hematoma formation or intimal disruption, resulting in compression of the true lumen and impaired coronary blood flow [3,7,8,9]. This pathophysiological mechanism differs fundamentally from plaque rupture-mediated thrombosis and necessitates distinct diagnostic and therapeutic considerations [5]. Advances in coronary angiography and intravascular imaging have improved recognition; however, SCAD remains underdiagnosed in cases presenting with subtle or atypical angiographic findings [7,8,10,11]. Accumulating observational evidence supports a predominantly conservative approach in hemodynamically stable patients, reflecting the high rates of spontaneous vessel healing and the technical challenges associated with percutaneous coronary intervention [3,4,5,9,12]. However, despite improvements in acute management, important gaps persist regarding long-term care. These evolving insights have reshaped contemporary management paradigms, emphasizing a pathophysiology-driven approach rather than routine revascularization. Despite favorable short-term survival, important gaps persist in long-term care. Recurrent SCAD, persistent chest pain syndromes, and psychosocial impairment have been increasingly reported during follow-up, underscoring that favorable short-term survival does not equate to benign long-term prognosis [13,14]. While prior reviews have extensively addressed the epidemiology, pathophysiology, and acute management, fewer have focused on structured follow-up strategies and imaging-guided long-term management. In particular, the role of non-invasive imaging modalities—including coronary computed tomography angiography (CCTA)—in confirming vessel healing and guiding clinical management continues to evolve. This review synthesizes current evidence on the epidemiology, pathophysiology, diagnosis, treatment, recurrence, and follow-up of SCAD, with particular emphasis on imaging-informed clinical decision-making and its implications for long-term management. Importantly, this review aims to highlight practical clinical decision-making and structured follow-up strategies while complementing a comprehensive overview of SCAD pathology.

## 2. Epidemiology

SCAD accounts for approximately up to 5% of all ACS cases in the general population; however, its relative contribution increases substantially in specific subgroups [15,16,17]. Approximately 90% of patients are women who present between 45 and 55 years of age. Among them, SCAD has been reported to account for 20–35% of myocardial infarctions, establishing it as a leading cause of non-atherosclerotic ACS in this population [5,18,19,20]. In contrast, male patients tend to present at a younger age and are more frequently associated with intense physical exertion as a precipitating factor, especially fibromuscular dysplasia [6,20,21,22]. Importantly, pregnancy-associated SCAD represents a distinct and clinically significant subgroup. Although it accounts for less than 15 % of cases, a minority of total SCAD cases, pregnancy-related SCAD disproportionately affects younger women and is often characterized by more proximal coronary involvement, including left main or multivessel disease [5,23,24,25]. Observational data suggest that pregnancy-associated SCAD may be associated with larger infarct size and increased short-term clinical severity, underscoring the need for heightened clinical vigilance in this population. From an anatomical perspective, SCAD most commonly involves the left anterior descending artery, accounting for approximately 40–50% of cases, followed by the circumflex and right coronary arteries [3,26]. Distal and mid-vessel segments are more frequently affected than proximal segments, a distribution that has important implications for both diagnosis and management [27]. Distal predominance may contribute to subtle angiographic findings and can limit the feasibility of percutaneous coronary intervention [11]. Multivessel SCAD is reported in a minority of patients, 9% to 23% of cases, but is associated with greater myocardial injury, more complex clinical presentation, and increased management challenges [21,28,29]. Longitudinal data further indicate that SCAD is not a uniformly benign condition. Long-term mortality after SCAD appears to be low, with estimates ranging from 1% over 3 years to approximately 2% within the first year [30,31]. Registry data suggest that although overall mortality after SCAD is low, nonfatal events remain clinically relevant. In the Canadian SCAD Registry, the 3-year MACE rate was 14%, driven mainly by recurrent myocardial infarction (10%), with comparatively lower rates of revascularization (3.5%) and mortality (1%) [32]. Similarly, the Australian–New Zealand SCAD cohort reported a 3-year MACE rate of 11.3%, with recurrent MI accounting for 5.7% of cases, while revascularization and heart failure occurred in 1.7% and 1.5%, respectively [2]. These epidemiologic characteristics—including demographic predilection, anatomical distribution, and recurrence risk—have direct implications for clinical surveillance strategies and long-term management planning.

## 3. Risk Factors

Although the precise etiology of SCAD remains unclear, it is thought to result from an interplay between underlying vascular susceptibility and precipitating triggers, including emotional stress, intense physical strain, stimulant medications or illicit drug use, and hormonal influences such as pregnancy [33]. Sex-related differences in reported triggers have been observed, with women more commonly describing antecedent emotional stress and men more frequently reporting physical stress, including aerobic and isometric exertion [34]. Consistent with this pathobiology, SCAD typically occurs in individuals with a low prevalence of traditional cardiovascular risk factors, with the notable exception of hypertension, which is reported in approximately 30% of cases [2,35]. In several nontraditional risk factors, fibromuscular dysplasia (FMD) is most notably identified in a substantial proportion of patients undergoing systematic vascular screening. In the cohorts with the highest incidence of screening, more than 50% of the patients also had FMD [30,36]. FMD emerged as an independent predictor of adverse outcomes and SCAD recurrence [34,37,38]. During long-term follow-up (median 21 months), it was associated with a higher risk of both major adverse cardiovascular events (HR 2.2, 95% CI 1.05–4.5, *p* = 0.037) and recurrence (HR 3.9, 95% CI 1.5–26.5, *p* = 0.01) [2]. Pregnancy-associated SCAD represents a distinct and clinically important subgroup [39]. Most cases occur during late pregnancy or the early postpartum period, suggesting that hormonal fluctuations and hemodynamic changes may contribute to increased arterial wall vulnerability [40,41]. With each pregnancy, additional risk factors may be introduced, and their cumulative impact may increase the long-term risk of SCAD, thereby serving as a potential predisposing factor for coronary artery dissection [42].

## 4. Pathophysiology

The pathophysiological mechanisms underlying SCAD remain incompletely understood. Several mechanisms have been proposed, including inflammatory processes, arterial tortuosity, intimal disruption, and spontaneous intramural hemorrhage [43,44,45,46]. In particular, two principal hypotheses have been advanced to explain intimal disruption and intramural bleeding: the “inside-out” and “outside-in” mechanisms. The former postulates that a disruption or tear of the intimal layer allows blood to enter the subintimal space, leading to the formation of a false lumen. Propagation of blood flow within this space contributes to expansion of the intramural hematoma and progressive luminal narrowing. In contrast, the “outside-in” hypothesis suggests that spontaneous rupture of the vasa vasorum within the media or adventitia results in intramural hematoma formation, which enlarges over time without an initial intimal tear [47]. Regardless of whether the intramural hematoma originates via an inside-out or outside-in mechanism, expansion of the hematoma ultimately compresses the true lumen and impairs coronary blood flow [48]. This reduction in coronary perfusion leads to myocardial ischemia and, in severe cases, myocardial infarction due to supply–demand mismatch. Emerging evidence indicates that genetic predisposition and molecular mechanisms may play a contributory role in the pathogenesis of SCAD. Recent integrative studies have identified associations between SCAD risk and genetic variants involved in connective tissue integrity, vascular biology, and extracellular matrix regulation. In particular, Ardissino et al. demonstrated that circulating proteins such as ECM1, AFAP1, SPON1, and STAT6 are genetically associated with SCAD susceptibility, implicating pathways related to extracellular matrix remodeling and inflammatory signaling, including the JAK–STAT pathway. Although these findings remain preliminary and require further validation, they underscore an evolving mechanistic framework that may enhance risk stratification and inform future targeted therapeutic strategies [49].

## 5. Clinical Presentation

In studies involving patients with SCAD, chest pain has been reported as the most common presenting symptom, occurring in approximately 90% of cases, with a frequency even higher than that observed in atherosclerotic myocardial infarction. Additional symptoms may include pain radiating to the arm, neck, or back, as well as nausea, vomiting, diaphoresis, and dyspnea [3,4,5,50]. Among the overall SCAD population, non-ST-segment elevation myocardial infarction (NSTEMI) is more common than ST-segment elevation myocardial infarction (STEMI), and SCAD is recognized as a frequent cause of myocardial infarction with non-obstructive coronary arteries (MINOCA) [2,51,52]. In approximately 0.5% of cases, SCAD may present as sudden cardiac death. Ventricular tachycardia and ventricular fibrillation have been reported in approximately 3.6% and 11.8% of patients, respectively. Cardiogenic shock has been observed in 1.2% to 15.9% of cases [27,53].

## 6. Diagnosis

**Figure 1:**
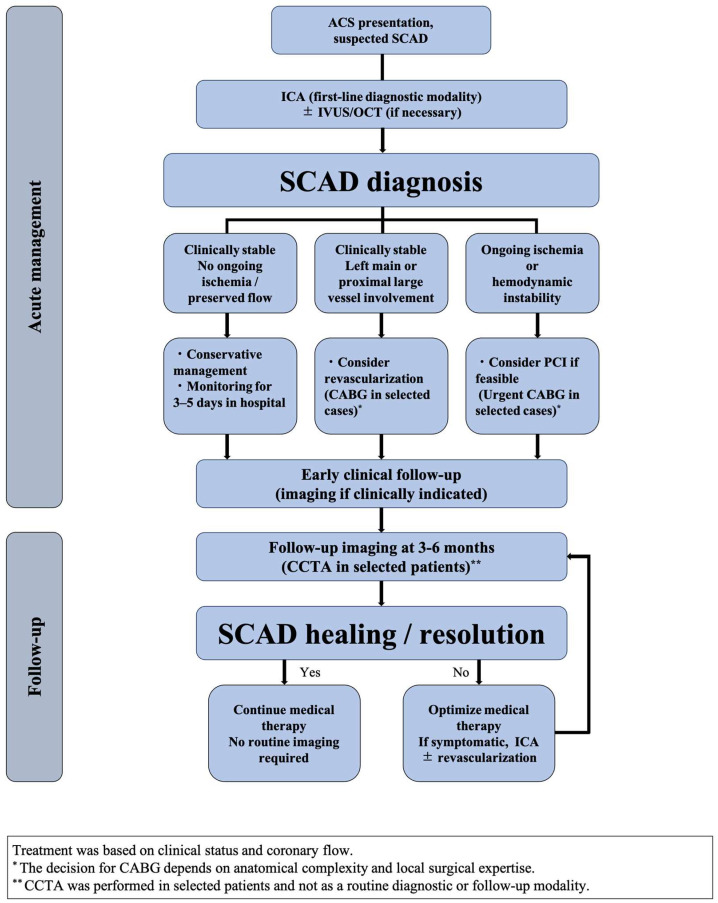
Flowchart illustrating diagnostic evaluation, acute management, and follow-up strategies in patients with SCAD, with CCTA used selectively in appropriate clinical contexts. The proposed algorithm represents a conceptual framework based on currently available evidence and should not be interpreted as a definitive guideline.

The management of SCAD differs substantially from that of typical atherosclerotic ACS, as a conservative strategy is generally favored over routine revascularization. Accordingly, accurate and timely diagnosis is essential [9]. To provide an overview of the diagnostic approach, acute-phase management, and follow-up strategy, a stepwise clinical flow is summarized in Figure 1. Evaluation of suspected SCAD should include a thorough clinical history and physical examination, particularly in patients belonging to higher-risk groups [54]. In the acute setting, invasive coronary angiography (ICA) remains the cornerstone for differentiating SCAD from other causes of ACS [5]. Heightened awareness of SCAD among interventional cardiologists in recent years has led to greater diagnostic recognition and an apparent increase in reported cases [55]. However, in cases where angiographic findings are inconclusive or atypical, intravascular imaging modalities such as intravascular ultrasound (IVUS) or optical coherence tomography (OCT) can provide additional diagnostic clarification by visualizing intramural hematoma or intimal disruption [56]. Intracoronary imaging modalities are not without risk, as guidewire or imaging catheter manipulation may propagate the dissection or cause iatrogenic injury, including guide-catheter-induced dissection. Therefore, their use should be reserved for selected cases in which the anticipated diagnostic benefit clearly outweighs the procedural risks [3,5,9,57].

### 6.1. Invasive Coronary Angiography and Intravascular Imaging

#### 6.1.1. Invasive Coronary Angiography

**Figure 2:**
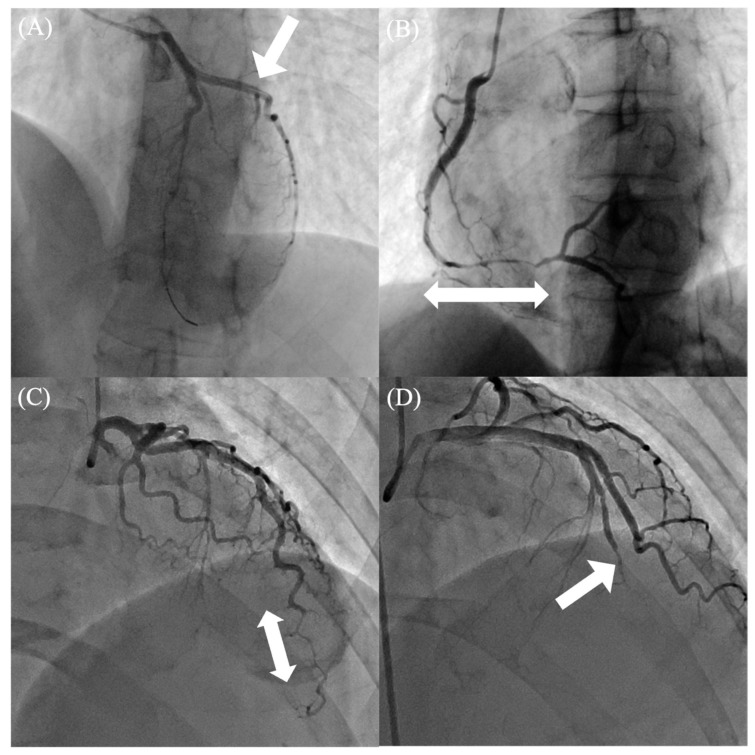
Angiographic features and classification of SCAD. (**A**) Type 1 SCAD involving the proximal LCX, in which the arrows indicate contrast staining of the arterial wall and the presence of multiple lumens. The lesion became apparent after guidewire insertion into the LAD, where SCAD was initially suspected. (**B**) Type 2 SCAD affecting the mid-RCA, in which the arrows indicate a long segment of diffuse, smooth luminal narrowing consistent with intramural hematoma. (**C**) Type 3 SCAD involving the distal LAD, in which the arrow indicates a focal stenosis that mimics atherosclerotic disease. (**D**) Type 4 SCAD presenting as complete occlusion (arrow) from the mid-LAD, which may be difficult to distinguish from thrombotic occlusion in the acute setting. Abbreviations: LAD = left anterior descending artery; LCX = left circumflex artery; RCA = right coronary artery; SCAD = spontaneous coronary artery dissection.

ICA remains the gold standard for the diagnosis of SCAD [3]. SCAD can occur in any coronary artery; however, the left anterior descending artery and its branches are most frequently involved [32]. Multivessel SCAD affecting non-contiguous coronary territories has been reported in approximately 10–15% of patients [58]. Angiographically, SCAD is categorized into four distinct types, primarily based on the presence or absence of an intimal tear [11], as illustrated by representative angiographic findings in Figure 2. Type 1 SCAD demonstrates the classic angiographic appearance of arterial dissection. Contrast enters both the true and false lumens through an intimal tear, resulting in multiple radiolucent lumens separated by a visible radiolucent flap. Contrast staining of the arterial wall and delayed clearance of contrast within the false lumen may also be observed (Figure 2A) [59]. Type 2 SCAD is the most common angiographic subtype, accounting for approximately 60–75% of cases. It is characterized by the absence of a visible intimal tear and presents as a long, diffuse, smooth narrowing of varying severity due to intramural hematoma. These lesions are typically longer than 20 mm and often appear as an abrupt change in vessel caliber (Figure 2B) [60]. Type 3 SCAD is less common and represents the most diagnostically challenging subtype (Figure 2C). Although also caused by intramural hematoma, type 3 lesions are typically shorter than 20 mm and may closely resemble atherosclerotic disease. Because of this similarity, additional diagnostic modalities are often required to establish an accurate diagnosis. This subtype should be suspected when there is a strong clinical suspicion of SCAD, absence of atherosclerosis in other coronary segments, a linear lesion of intermediate length (approximately 11–20 mm), or the presence of marked coronary tortuosity [61]. When type 2 or type 3 SCAD is suspected, intracoronary nitroglycerin administration during angiography is recommended to exclude coronary vasospasm as a potential cause of the angiographic abnormalities [9,11]. Finally, type 4 SCAD is characterized by complete vessel occlusion (Figure 2D). Its angiographic appearance may mimic thromboembolic occlusion. In such cases, the underlying dissection may only become evident after spontaneous recanalization or repeat angiography demonstrates vessel healing once embolic causes have been excluded [62,63,64].

#### 6.1.2. Intravascular Imaging

Both IVUS and OCT contribute to the identification and delineation of intramural hematoma and may also assist in risk stratification and therapeutic decision-making, including the assessment of the absence of atherosclerotic plaque suggestive of typical coronary artery disease [65]. However, the precise role of intravascular imaging within the diagnostic algorithm for SCAD has not yet been clearly established, and safety data in this patient population remain limited [3,59]. In the Canadian SCAD Cohort Study, which included 1306 patients with SCAD, procedural complications related to intracoronary imaging were reported in 8.6% of cases in which such imaging was performed [66]. When intravascular imaging is undertaken—particularly in patients for whom a conservative management strategy is anticipated—it is recommended that the examination be limited to what is necessary to confirm the diagnosis and demonstrate the presence of intramural hematoma.

##### IVUS

**Figure 3:**
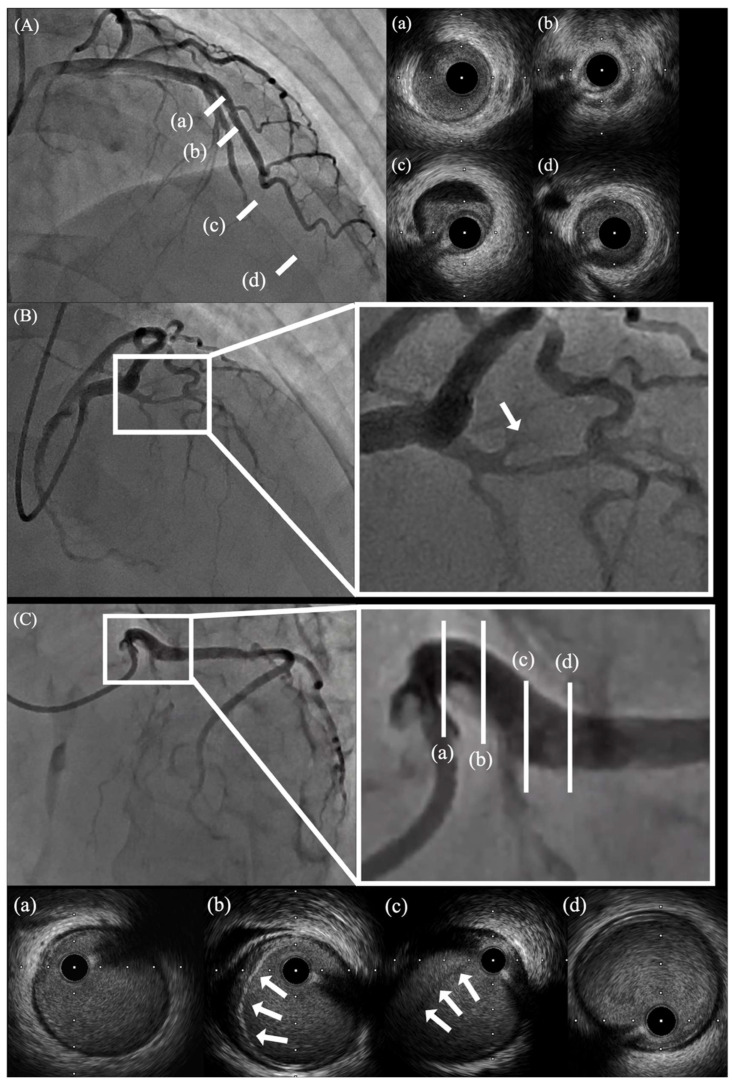
Intracoronary imaging: IVUS. (**A**) IVUS findings of the LAD in a case of type 4 SCAD. (**a**) No evidence of dissection proximal to the first septal branch. (**b**,**c**) Luminal narrowing caused by intramural hematoma. (**d**) Distal segment without extension of the dissection. (**B**,**C**) IVUS findings from the same case demonstrating type 4 SCAD extending from the proximal LAD. (**B**) The arrow indicates the site of occlusion. (**C**) IVUS images obtained from the LCX toward the LMT. (**a**) No abnormal findings in the mid-LMT. (**b**) The distal LMT represents the entry point of the dissection (arrow), showing a false lumen separated by an intimal flap. (**c**) At the bifurcation from the LMT into the LAD and LCX, the left side of the image corresponds to the LAD, demonstrating a false lumen and intramural hematoma separated by a flap (arrow), resulting in compression and narrowing of the true lumen of the LAD. (**d**) No extension of the dissection into the LCX. Abbreviations: IVUS = intravascular ultrasound; LMT = left main trunk.

IVUS does not require contrast injection and allows visualization of intramural hematoma and false lumen formation [67]. IVUS can distinguish SCAD from atherosclerotic plaque in many cases and clearly delineate true and false lumens, including the extent of false lumen thrombosis [68], as illustrated in representative SCAD cases in Figure 3. A major advantage of IVUS over OCT is its superior depth penetration (approximately 4–8 mm), enabling full-thickness visualization of the arterial wall up to the external elastic membrane. This is particularly useful in large vessels and in cases with extensive longitudinal dissection, as IVUS can capture the proximal and distal margins of intramural hematoma [11,69]. Additionally, because blood clearance is not required, IVUS eliminates the risk of contrast-induced hydraulic propagation of dissection and allows continuous real-time monitoring during interventional procedures [70,71]. However, IVUS has limitations. Its lower spatial resolution compared with OCT may hinder identification of small intimal tears, thin residual intimal flaps, or subtle communications between true and false lumens [64,72]. Grayscale imaging may also make it difficult to distinguish intramural hematoma from lipid-rich atherosclerotic plaque, particularly in less experienced hands [71]. Furthermore, the catheter profile may limit access to distal or highly tortuous coronary segments and carries a potential risk of iatrogenic injury [73].

##### OCT

**Figure 4:**
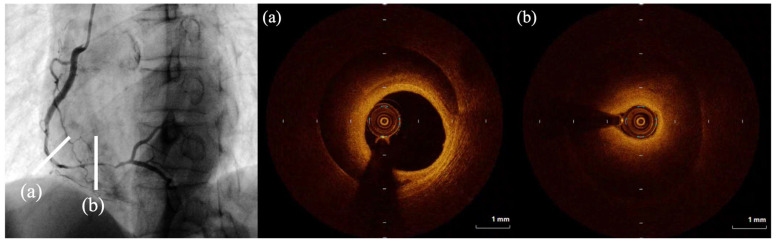
Intracoronary imaging: OCT. OCT findings of a type 2 SCAD lesion in the mid-RCA. (**a**) Intramural hematoma is present with preserved luminal integrity. (**b**) Luminal narrowing caused by intramural hematoma.

Optical coherence tomography (OCT) provides high-resolution imaging (approximately 10–20 μm, nearly tenfold higher than IVUS) using near-infrared light delivered through an optical fiber catheter. This high spatial resolution allows detailed visualization of coronary arterial wall layers (intima, media, and adventitia) and identification of subtle structural abnormalities, including small intimal tears, thin residual intimal membranes between true and false lumens, intramural thrombus, and inflammatory infiltration [74], as illustrated in representative SCAD cases evaluated by OCT in Figure 4. In SCAD, OCT clearly delineates double-lumen morphology and the boundaries of intramural hematoma, and it can precisely identify the entry point of intimal disruption when present [75]. OCT also enables accurate measurement of luminal dimensions and vessel wall thickness, which may be helpful when stent sizing is required during percutaneous intervention [76,77]. Despite its superior resolution, OCT has important limitations. Because near-infrared light does not penetrate blood, contrast injection is required to clear the lumen during image acquisition. This necessity raises concerns regarding hydraulic extension of dissection due to contrast jet injection [78]. Additionally, OCT has limited tissue penetration depth (approximately 1–3 mm), which may restrict evaluation of large intramural hematomas or full vessel wall thickness in larger arteries [79]. The imaging window is also relatively short, as rapid catheter pullback over a few seconds is required, preventing continuous real-time monitoring of long segments [3,9,69].

### 6.2. Non-Invasive Imaging Modalities

#### 6.2.1. Coronary Computed Tomography Angiography

Coronary computed tomography angiography (CCTA) has emerged as a non-invasive imaging modality with potential diagnostic utility in selected cases of suspected SCAD [80]. Although ICA remains the diagnostic gold standard, several recent studies have systematically evaluated the diagnostic performance and anatomical characterization of CCTA in acute SCAD. In a case–control study by Moser et al., including 52 patients with angiographically confirmed acute SCAD and 70 matched controls without SCAD, the diagnostic performance of CCTA was systematically assessed. At the patient level, CCTA demonstrated a sensitivity of 52–58% and a specificity of 97%, with overall diagnostic accuracy of 78–80%. When myocardial hypoattenuation was incorporated as an additional diagnostic criterion, sensitivity improved (up to 65–71%) at the expense of reduced specificity (91%). These findings indicate that while CCTA has high rule-in capability, its sensitivity—particularly for subtle or distal dissections—remains moderate [41,81]. Similarly, in a prospective multicenter study by Pagonis et al., patients with angiographically confirmed SCAD underwent CCTA within a mean of 3.2 days after ICA. At the segmental level, agreement between ICA and CCTA for dissection detection was moderate (κ = 0.60 overall). Agreement was higher in proximal segments (κ = 0.74) but declined in distal segments (κ = 0.57), reflecting reduced spatial resolution in small-caliber vessels. Of 46 dissected segments identified on ICA, only 22 were detected on CCTA [82]. These data reinforce the limitation of CCTA in distal vessel involvement, which is common in SCAD. Typical CCTA findings in acute SCAD include crescent-shaped mural thickening corresponding to intramural hematoma, smooth long-segment luminal narrowing, abrupt or tapered changes in vessel caliber, linear low-attenuation areas suggestive of a false lumen, and the absence of calcified or lipid-rich atherosclerotic plaque [15]. A systematic imaging review further emphasized that SCAD lesions frequently demonstrate negative remodeling, diffuse wall thickening, and lack of high-risk plaque features such as napkin-ring sign or spotty calcification, which are more characteristic of atherosclerotic disease. Therefore, assessment of plaque burden—or its absence—represents an important adjunctive element in the differential diagnosis [83,84]. Despite its anatomical advantages, CCTA is constrained by spatial resolution limitations (approximately 500–625 μm), compared with invasive angiography (~160 μm) and intracoronary imaging [85]. These technical differences explain the reduced detection of small intimal tears and distal dissections. Additional limitations include reduced sensitivity in small-caliber (<2.0 mm) or distal vessels, difficulty differentiating type 4 SCAD from thromboembolic occlusion, susceptibility to motion artifacts in patients with elevated heart rates, and limited ability to detect subtle intimal flaps without overt intramural hematoma [84,85,86]. Accordingly, a negative CCTA does not exclude SCAD, particularly in distal or limited disease. A large U.S. database study including 11,052 patients hospitalized with ICA-confirmed SCAD between 2017 and 2022 reported that only 1.6% underwent CCTA during hospitalization. However, utilization increased modestly over time (1.4% in 2017 to 2.1% in 2022). Patients undergoing CCTA were generally younger and less likely to undergo PCI, suggesting that CCTA may be selectively employed in more stable patients or in cases managed conservatively [87]. Taken together, current evidence indicates that CCTA demonstrates high specificity but only moderate sensitivity for the diagnosis of acute SCAD, particularly in distal or small-caliber vessels. Therefore, CCTA should not replace ICA in the acute setting. Unlike ICA, coronary CT angiography does not require catheter instrumentation of the coronary arteries and thus avoids the risk of iatrogenic propagation of an existing dissection [88]. In carefully selected, hemodynamically stable patients—particularly when angiographic findings are equivocal, when differentiation from atherosclerotic disease is necessary, or when detailed anatomical characterization may influence management decisions—CCTA may serve as a complementary diagnostic tool. Therefore, its role should be interpreted cautiously and is best considered in the follow-up setting rather than as a first-line diagnostic approach.

#### 6.2.2. Cardiac Magnetic Resonance

In patients with suspected SCAD, cardiac magnetic resonance (CMR) is used primarily for the assessment of myocardial injury rather than for direct visualization of the coronary dissection itself [89]. The presence of late gadolinium enhancement (LGE) in a distribution corresponding to the culprit coronary territory suggests ischemic myocardial injury and may support the diagnosis. In contrast, a non-ischemic pattern of LGE may indicate alternative diagnoses, such as myocarditis [4]. However, CMR may be normal in cases of transient ischemia or limited myocardial injury; therefore, a normal CMR does not exclude SCAD [70]. In recent years, CMR parameters have also emerged as potential tools for prognostic risk stratification in SCAD. In a prospective single-center study including 59 patients with angiographically confirmed SCAD, CMR was performed at a median of 4 days after symptom onset. Follow-up evaluation was conducted at a mean of 5.5 months after discharge, and the presence of more than three transmural LGE segments independently predicted major adverse cardiac events (hazard ratio, 1.50; *p* = 0.006) [90].

## 7. Treatment

### 7.1. Acute-Phase Management

SCAD in the acute setting should not be managed solely on the basis of angiographic findings; rather, therapeutic decisions should be guided primarily by hemodynamic stability, the extent of myocardial ischemia, and coronary flow. Unlike atherosclerotic ACS, SCAD is frequently associated with a high rate of spontaneous vessel healing. This fundamental difference underlies the principle of prioritizing a conservative strategy in appropriately selected patients [3,4,5]. Therefore, individualized treatment tailored to clinical severity and risk profile, grounded in a conservative-first approach, is essential.

#### 7.1.1. Hemodynamically Stable: Conservative-First Strategy

In hemodynamically stable patients without ongoing ischemia and with preserved or acceptable distal coronary flow, conservative management is generally preferred [91]. Observational data consistently demonstrate high rates of spontaneous angiographic healing—often within 1 to 3 months—supporting a non-interventional approach in most cases [92]. In the DISCO Registry (n = 370), 65% of patients were managed with an initial conservative strategy [93]. Likewise, in the Canadian SCAD (CanSCAD) Registry (n = 750), 86% of patients were treated conservatively as the initial approach [28]. Furthermore, the study by Krittanawong et al. demonstrated that, in the majority of SCAD patients, optimal medical therapy (OMT) was associated with lower mortality and complication rates compared with PCI [94]. Collectively, these findings clearly illustrate a paradigm shift in SCAD management over time, with an increasing preference for conservative treatment rather than routine revascularization [12]. Conservative management includes close clinical monitoring during acute hospitalization, optimization of blood pressure, and initiation of appropriate medical therapy [46]. Repeat invasive angiography is not routinely indicated in clinically stable patients, given the potential risk of iatrogenic extension of dissection. Instead, careful observation, imaging on follow-up tailored to each patient’s needs, and symptom-guided reassessment are recommended [6]. 

#### 7.1.2. Hemodynamically Unstable or Ongoing Ischemia: Revascularization Strategy

In contrast, invasive revascularization should be considered in patients with hemodynamic instability or cardiogenic shock, ongoing or recurrent ischemia, left main coronary artery involvement, more than two instances of proximal large-vessel occlusion with extensive myocardial jeopardy, and severely impaired coronary flow [3]. In such high-risk scenarios, the potential benefit of restoring coronary perfusion may outweigh procedural risks. Patients who underwent revascularization as the initial treatment strategy were shown to have a higher risk of recurrent SCAD in the same vessel compared with those managed conservatively as the first-line approach [95]. Therefore, careful assessment of the potential risks remains essential before proceeding with revascularization.

##### PCI

PCI remains the most frequently employed revascularization strategy; however, it is associated with higher technical complexity compared with atherosclerotic disease. Challenges include dissection extension, intramural hematoma propagation, difficulty in confirming true lumen wire position, and the need for extensive stent coverage [96]. Data from the Canadian SCAD Cohort Study demonstrated that PCI was attempted in approximately 30% of patients, with procedural success rates ranging from 50–70%, substantially lower than in atherosclerotic disease. Procedural complications—including propagation of dissection, extension of intramural hematoma, and the need for unplanned additional stenting—were reported in up to 30–40% of cases [28]. Similarly, observational registry data indicate that long stent implantation is frequently required due to the longitudinal extension of intramural hematoma, increasing the risk of late stent malapposition after vessel healing [97]. In a prospective cohort study of 403 patients with SCAD, those who underwent PCI had significantly higher rates of in-hospital adverse events and major adverse cardiovascular events (MACEs) compared with patients managed conservatively [98]. In a comparison of recurrence rates between an initial revascularization strategy and conservative management, SCAD recurrence in the index vessel was observed among patients treated with PCI, while no recurrence in the index vessel occurred in patients managed conservatively [21]. Taken together, PCI in SCAD represents a carefully balanced decision: while life-saving in high-risk presentations, it should be reserved for clearly defined indications and performed with a flow-oriented, minimalistic strategy under experienced operators.

##### CABG

The role of coronary artery bypass grafting (CABG) in the management of SCAD is extremely limited and should be considered only as a last-resort strategy in rare circumstances. Fewer than 1% of patients with acute SCAD are referred for surgical revascularization [28]. Such situations include left main coronary artery dissection or extensive multivessel dissection in which percutaneous intervention is not feasible or is unlikely to achieve an acceptable result. CABG may also be considered as a salvage strategy in cases of failed PCI or persistent complete occlusion of a major coronary artery [9]. In particular, surgical revascularization may be warranted when PCI is technically challenging and the ischemic territory is extensive, rendering medical therapy alone insufficient to relieve ongoing myocardial ischemia [46]. Conduit selection should be guided by the high likelihood of spontaneous healing of the native vessel and the risk of competitive flow, which may lead to late graft failure. Competitive flow from a recanalized native coronary artery represents a major concern in SCAD, as it increases the risk of graft occlusion. Therefore, saphenous vein grafts may be more appropriate for targets other than the left anterior descending artery, where long-term graft patency is generally considered less critical [99].

### 7.2. Pharmacotherapy (Acute to Discharge and Beyond)

Pharmacologic therapy plays a central role in SCAD management and is typically initiated irrespective of whether the initial strategy is conservative or invasive. However, therapeutic decisions should reflect the distinct pathophysiology of SCAD rather than extrapolate directly from atherosclerotic ACS paradigms. 

#### 7.2.1. β-Blockers

By reducing heart rate and arterial shear stress, beta-blockers may mitigate mechanical forces implicated in dissection propagation. Observational studies suggest an association between beta-blocker use and reduced recurrence risk [31,100]. In the absence of contraindications, long-term continuation is generally recommended.

#### 7.2.2. Antiplatelet Therapy

Antiplatelet therapy in SCAD remains an area of active investigation. In patients undergoing PCI with stent implantation, DAPT should follow contemporary PCI guidelines. In conservatively managed patients, the optimal antiplatelet strategy is less clearly defined. Approximately 90% of patients are discharged on at least one antiplatelet agent, with aspirin monotherapy being widely used [18]. In patients with SCAD treated conservatively, aspirin therapy is generally advised for at least 12 months and is frequently continued long term unless contraindicated [101]. In patients with SCAD managed conservatively as the initial treatment strategy, observational data suggest that dual-antiplatelet therapy (DAPT) is associated with higher rates of major adverse cardiovascular events (MACEs) at 12 months compared with single-antiplatelet therapy (SAPT) [102]. However, robust evidence supporting the benefit of prolonged DAPT in patients without stent implantation remains lacking. Therefore, the importance of individualized treatment decisions, balancing potential ischemic benefit against bleeding risk, is emphasized.

#### 7.2.3. Anticoagulation

Given that SCAD is primarily driven by intramural hematoma rather than luminal thrombus, routine continuation of anticoagulation after diagnosis is generally discouraged unless there is another compelling indication [103]. Thrombolytic therapy is contraindicated in most SCAD cases due to the potential risk of hematoma expansion and clinical deterioration [97].

#### 7.2.4. Statins

Unlike atherosclerotic ACS, routine statin therapy is not universally indicated in SCAD unless there is coexisting dyslipidemia or established atherosclerotic disease. Current evidence does not support universal statin prescription solely on the basis of SCAD diagnosis [3].

#### 7.2.5. Antianginals

Chest pain following SCAD is common and represents a frequent cause of hospital readmission, accounting for approximately 20% of readmissions within 30 days after SCAD-related acute myocardial infarction [104]. Chest pain may persist for several months even when ischemia testing is normal or repeat coronary imaging demonstrates vessel healing [105]. In patients with ongoing atypical chest pain without evidence of ischemia on functional testing, alternative mechanisms should be considered, including coronary vasospasm, endothelial dysfunction, microvascular disease, menstrual-related chest pain, and non-cardiac causes. Nitrates and calcium channel blockers may be helpful in alleviating symptoms [3].

### 7.3. Chronic-Phase Management

The mortality rate after SCAD is low, reported at approximately 1% at three years and 2% at one year. In contrast, the recurrence rate of myocardial infarction related to SCAD is relatively high, reaching approximately 17–18% within 3 to 4 years of follow-up [5]. Long-term management of SCAD extends beyond the acute event and requires structured, multidisciplinary care focused on recurrence prevention, symptom management, and psychosocial well-being. 

#### 7.3.1. Long-Term Blood Pressure Control

Hypertension has been identified as a factor associated with an increased risk of SCAD recurrence [31]. Sustained blood pressure optimization remains a cornerstone of chronic management. Beta-blockers are often continued long term, and additional antihypertensive agents may be introduced as needed to achieve individualized targets.

#### 7.3.2. Cardiac Rehabilitation

Individualized exercise prescription and participation in a structured cardiac rehabilitation (CR) program constitute a cornerstone of chronic disease management in SCAD survivors. Multiple studies have demonstrated the safety and efficacy of CR in this population [106]. Importantly, the level of physical activity at the time of SCAD onset does not appear to predict the risk of future adverse events [54]. Patients are generally advised to engage in moderate-intensity aerobic exercise combined with low-resistance training while avoiding extreme isometric exertion and high-endurance competitive sports [107].

#### 7.3.3. Psychosocial and Mental Health Support

Migraine, anxiety, depression, and post-traumatic stress disorder (PTSD) are commonly observed among patients following SCAD and may substantially impair quality of life [108]. Appropriate screening, timely treatment, and referral to mental health services are therefore recommended. Individualized counseling is essential, including education on symptom recognition, avoidance of potential triggers, and optimization of physical activity levels [3]. In particular, patients with peripartum SCAD are at heightened risk of PTSD, anxiety, and depression, as they must cope not only with physical recovery but also with the new responsibilities of motherhood. Comprehensive multidisciplinary support is therefore crucial in this vulnerable population [40].

#### 7.3.4. Assessing for Concomitant Fibromuscular Dysplasia

Because patients with SCAD frequently have concomitant extracoronary arterial abnormalities, comprehensive axial vascular imaging from the head to the pelvis is recommended [109]. In particular, given the high prevalence of fibromuscular dysplasia (FMD) in this population and its association with an increased risk of long-term major coronary events, whole-body vascular evaluation using computed tomography angiography (CTA), when feasible, is recommended [38].

## 8. Imaging-Based Follow-Up on CCTA

**Figure 5:**
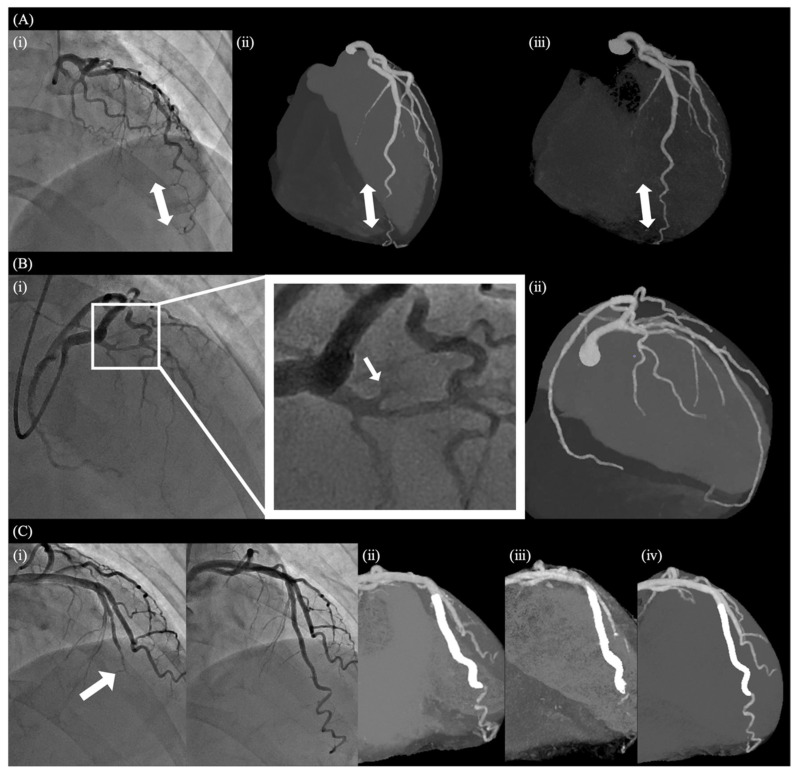
Follow-up CCTA after ICA or treatment in SCAD. (**A**) Type 3 SCAD in the distal LAD. (**i**) Arrow indicates the site of the lesion on admission. (**ii**) At 1-month follow-up CCTA after ICA, the dissection persists (arrow). (**iii**) Healing is observed at 7-month follow-up (arrow). (**B**) Type 4 SCAD in the proximal LAD. (**i**) Arrow denotes the occlusion site at presentation. (**ii**) CCTA performed 1 week after ICA demonstrates restoration of flow in the previously occluded vessel. (**C**) A case of type 4 SCAD in the mid-LAD. (**i**) Arrow marks the lesion site at presentation. The lesion was treated with PCI and DES implantation. Residual dissection is observed distal to the stent. (**ii**–**iv**) Serial CCTA images for follow-up of the lesion. At 1 week after PCI (**ii**) and at 3 months after PCI (**iii**), residual dissection persists distal to the stent. (**iv**) At 8 months after treatment, progressive healing is observed, with resolution of the dissection. Abbreviations: ICA = invasive coronary angiography; CCTA = coronary computed tomography angiography; PCI = percutaneous coronary intervention; DES = drug-eluting stent.

The natural history of SCAD is characterized by a high rate of spontaneous vessel healing [3,9]. Accordingly, imaging-based follow-up should be structured along a temporal framework that reflects this dynamic vascular remodeling process. In this context, CCTA is discussed primarily from a longitudinal management perspective, with emphasis on its role in guiding follow-up assessment and supporting individualized patient care. Following diagnostic ICA, CCTA is increasingly performed within the early post-diagnosis period to non-invasively document coronary morphology [88]. This early imaging serves primarily as a reference study, allowing visualization of intramural hematoma, luminal narrowing, vessel caliber changes, and coronary tortuosity [83]. Figure 5 illustrates representative cases of CCTA-based follow-up after ICA or treatment, demonstrating the temporal evolution of SCAD lesions, including persistence of dissection in the early phase, restoration of coronary flow, and subsequent vessel healing over time. These examples highlight the utility of CCTA for non-invasive assessment of vessel patency, residual dissection, and longitudinal vascular remodeling, as well as for evaluating the response to medical therapy. The emerging role of CCTA in SCAD reflects technical advances in spatial resolution and quantitative assessment, which enhance visualization of non-atherosclerotic arterial wall pathology [110]. Establishing a non-invasive baseline may be particularly useful in conservatively managed patients, where longitudinal comparison is anticipated. A structured follow-up strategy represents a key component of SCAD management, particularly in conservatively managed patients in whom spontaneous healing is anticipated. In clinical practice, residual coronary abnormalities on early imaging may contribute to persistent patient anxiety, especially in younger individuals and in those with stress-related triggers. In this context, non-invasive follow-up imaging such as CCTA may provide not only anatomical information on vessel healing but also clinically meaningful reassurance. Visualization of vessel recovery may support patient understanding of the disease course and facilitate psychological stabilization, which is an important but often underrecognized aspect of SCAD management. Healing of SCAD is time-dependent. Observational data suggest that most conservatively managed lesions demonstrate substantial resolution within several months [5]. In a prospective registry, CCTA performed at 3–6 months demonstrated complete healing in 83% of patients [111]. Similarly, CTCA follow-up data indicate that imaging performed ≥80 days after the index event improves diagnostic accuracy for healing (sensitivity 76.9%, specificity 84%) [112]. These findings support a delayed imaging strategy—typically around 3–6 months—when anatomical confirmation of recovery is clinically relevant. Comparison with early baseline imaging allows objective documentation of vessel remodeling and resolution of intramural hematoma. CCTA should not be considered mandatory for all SCAD patients. However, within a structured follow-up strategy, it represents a reasonable and pragmatic non-invasive modality in selected scenarios, including persistent or recurrent chest pain, uncertainty regarding vessel healing, pre-return-to-activity evaluation, and situations where anatomical confirmation may influence management. Importantly, CCTA avoids the potential risks of repeat invasive angiography, including propagation of dissection or iatrogenic vessel injury [2]. Limitations remain, particularly reduced sensitivity for distal or small-caliber coronary segments, where many SCAD lesions occur [3]. Therefore, imaging decisions should be individualized based on initial anatomy and clinical context. In clinical practice, if follow-up imaging at approximately 3–6 months demonstrates limited or incomplete vessel healing compared with baseline findings, intensification of medical therapy and closer surveillance may be considered. In such cases, repeat CCTA at a later time point can be contemplated to reassess vascular remodeling. Although high-quality prospective evidence supporting a standardized imaging interval is currently lacking, this stepwise approach may be reasonable in selected patients within an individualized management framework, particularly in patients with incomplete healing or persistent symptoms. It should also be noted that most current management strategies are primarily based on observational data and expert consensus, and comparative evidence remains limited, underscoring the need for further prospective investigation. This underscores the importance of individualized clinical judgment in current practice, particularly in integrating imaging findings into follow-up decision-making in the context of limited evidence.

## 9. Conclusions

SCAD is a distinct cause of ACS requiring a management strategy fundamentally different from atherosclerotic disease. Advances in invasive and non-invasive imaging have improved diagnostic recognition and clarified its unique pathophysiology. Current evidence supports a conservative-first approach in hemodynamically stable patients, reflecting high rates of spontaneous vessel healing and the technical complexity of revascularization. Although long-term mortality is low, SCAD is not benign. Recurrent myocardial infarction and persistent symptoms underscore the need for structured longitudinal care beyond the acute phase. Management should therefore integrate clinical stability, coronary anatomy, recurrence risk, and patient-specific factors within an individualized framework. In this evolving paradigm, imaging plays an essential role in both diagnosis and follow-up. Coronary computed tomography angiography has emerged as a valuable non-invasive tool for selected patients, particularly for documenting vessel healing and informing management decisions. While standardized follow-up algorithms remain undefined, a risk-adapted, patient-centered strategy is currently most appropriate. Further research is needed to refine imaging intervals, improve risk stratification, and optimize long-term therapeutic strategies. Until then, individualized, physiology-guided care remains central to optimal SCAD management.

## Data Availability

No new data were created or analyzed in this study.
